# Clinical and biochemical evaluation of children with short stature in the primary care setting: a systematic review

**DOI:** 10.1186/s13052-026-02215-8

**Published:** 2026-02-21

**Authors:** Chiara Mameli, Rino Agostiniani, Giuseppe Banderali, Elena Bozzola, Vita Antonella Di Stefano, Luigi Greco, Carmine Pecoraro, Simone Rugolotto, Sara Sollai, Elvira Verduci, Liliana Guadagni, Claudio Lo Giudice, Gaetano Caforio, Annamaria Staiano

**Affiliations:** 1 Department of Pediatrics, Vittore Buzzi Children’s Hospital, Università di Milano, Milan, Italy; 2 Department of Biomedical and Clinical Science, Università di Milano, Milan, Italy; 3 Department of Pediatrics, San Jacopo Hospital, Pistoia, Italy; 4 Department of Pediatrics ASST Santi Paolo e Carlo Hospital, Department of Health Science, Università di Milano, Milan, Italy; 5 Pediatric Unit, IRCCS Bambino Gesù Children Hospital, Rome, Italy; 6 Department of Pediatrics, Azienda Ospedaliera per l’Emergenza Cannizzaro, Catania, Italy; 7 General Pediatrician, ASST Papa Giovanni XXIII - Bergamo, Bergamo, Italy; 8 Pediatric Nephrology and Dialysis Unit, A.O.R.N. Santobono Pausilipon Children’s Hospital, Naples, Italy; 9 Division of Pediatrics and Neonatology, Department of Maternal Infant Medicine, Santa Maria della Misericordia Hospital, Rovigo, Italy; 10 Department of Pediatrics, Santa Maria Annunziata Hospital, Florence, Italy; 11 Department of Health Sciences, University of Milano, Milano, Italy; 12 Department of Surgical and Biomedical Sciences, University of Perugia, Perugia, Italy; 13 C.R.E.A. Sanità (Centre for Applied Economic Research in Healthcare), Rome, Italy; 14 Department of Translational Medical Sciences-Section of Pediatrics, University of Naples “Federico II”, Naples, Italy

## Abstract

Short stature is a frequent reason for pediatric referral, yet clear diagnostic criteria remain elusive. Variability in clinical definitions, reference growth charts, and laboratory screening tests complicates the evaluation of affected children. This systematic review aimed to analyze the diagnostic approaches used in clinical and biochemical assessments of short stature in children in the primary care setting. This systematic review was conducted in accordance with PRISMA 2020 guidelines and registered in PROSPERO (CRD420251002215). Two independent literature searches were performed to address two domains: clinical and biochemical assessment. Studies were selected based on predefined inclusion and exclusion criteria, and risk of bias was assessed using the JBI critical appraisal tools. All procedures were conducted by independent reviewers, and discrepancies were resolved by consensus or through a third reviewer. A total of 424 studies were included, 35 in the clinical and 7 in the biochemical domain. Definitions of short stature considered in the studies varied considerably: 28 studies used a threshold of < 2 standard deviation score, while 19 applied percentile-based cut-offs (mostly the 3rd percentile); 8 studies considered both definitions, 3 studies do not report any definition. Growth velocity and target height were rarely used, despite their diagnostic value. Local growth charts were employed when available; otherwise, WHO and CDC references were most used. Laboratory assessments also varied greatly, with complete blood count and urine/stool analyses commonly performed, while thyroid function and celiac disease screening were inconsistently applied. There is substantial heterogeneity in the clinical and biochemical evaluation of children with short stature across studies. This reflects the absence of universally accepted diagnostic criteria and standardized screening protocols. These findings emphasize the need for internationally accepted, evidence-based guidelines to improve diagnostic accuracy and clinical management of children with short stature.

**Supplementary Information:**

The online version contains supplementary material available at 10.1186/s13052-026-02215-8.

## Background

Short stature is a common reason for referral to pediatricians with a significant overlap between physiology and pathology with the former largely predominant over the latter. Various factors - including genetic, prenatal, postnatal, and environmental factors – can affect growth [1]. While most cases of short stature represent non-pathological variants of growth, growth failure may be the manifestation of underlying diseases. These include endocrine and genetic conditions, as well as systemic disorders, such as celiac disease and inflammatory bowel diseases, which may present with growth failure before other symptoms manifest [2].

Therefore, it is of outstanding importance to provide a correct assessment of children with short stature during screening with general practitioners for referral or at specialist evaluation with pediatric endocrinologist.

To the best of our knowledge, there is no international consensus on the criteria for defining normal and pathological growth in the clinical and biochemical evaluation of short stature in children, particularly regarding priority target conditions for growth screening. Scientific societies and special interest groups have produced different clinical standards for the initial assessment, investigation and testing of children with short stature. Most of these standards have been achieved with consensus based on expert opinions [3–5]. Only one guideline was published by Wit and collaborators using the PICO model [6].

The lack of international agreement and shared pathway may be explained by a significant heterogeneity among studies concerning the target population, the choice of growth charts, and the parameters used (e.g., height, growth velocity, target height) to define short stature. Additionally, differing cut-off points (e.g., percentiles vs. standard deviations) further contribute to this variability.

Due to the lack of consensus, we decided to conduct a systematic review of published peer-reviewed articles focusing on the clinical and biochemical evaluation of children with short stature. This systematic review examines diagnostic approaches to short stature in children, focusing on definitions, utilized reference growth charts, and the laboratory tests employed in pediatric primary care.

## Material and methods

A systematic review was performed in accordance with the PRISMA 2020 guidelines [7]; The review protocol was registered in the PROSPERO database (CRD420251002215) and was developed to address two different research questions:

1. What are the most reliable clinical criteria for diagnosing short stature in children?
2. What is the diagnostic accuracy and utility of commonly used laboratory tests in identifying the underlying causes of short stature?

Consequently, two separate literature searches were performed, one focusing on the clinical domain, and the other on the biochemical domain.

We selected studies according to the following inclusion and exclusion criteria.

### Population

Included

- Children referred for evaluation of short stature in a primary care setting.

Excluded

- Adults (>18 years old).
- Children with known chronic diseases that are already established causes of growth failure, such as: chronic kidney disease; congenital or acquired heart disease; cystic fibrosis; inflammatory bowel disease (e.g., Crohn’s disease, ulcerative colitis); untreated celiac disease (but studies including newly diagnosed celiac disease as part of the differential diagnosis may be considered); severe malnutrition with an identified etiology; ongoing oncological conditions (e.g., leukemia, brain tumors, history of cranial irradiation).
- Children with pre-diagnosed genetic conditions associated with short stature, such as: Turner syndrome, Noonan syndrome, Prader-Willi syndrome, Russell-Silver syndrome, achondroplasia and skeletal dysplasia.

### Intervention(s) or exposure(s)

#### Clinical examinations

- Physical examination to assess growth patterns and identify signs of syndromic or systemic conditions.
- Personal and family history to investigate prenatal, perinatal, and postnatal growth factors, as well as familial growth trends.

#### Auxological assessment

- Anthropometric measurements, including: height (standing or supine, depending on age), weight, head circumference, Body Mass Index (BMI), sitting height, arm span.
- Growth velocity assessment calculated using serial height measurements over a period of 6–12 months.
- Comparison with reference growth charts (Cacciari charts, INeS WHO, CDC,) to determine deviation from expected growth patterns.

#### Laboratory tests

- Growth hormone axis evaluation: IGF-1, IGFBP-3, growth hormone stimulation tests (if indicated).
- Thyroid function tests: TSH, free T4.
- Metabolic and biochemical panel: blood glucose, creatinine, electrolytes (sodium, potassium), calcium, phosphorus, alkaline phosphatase.
- Liver and kidney function tests: AST, ALT, GGT.
- Inflammatory and nutritional markers: complete blood count (CBC), C-reactive protein (CRP).
- Celiac disease screening.
- Venous blood gas analysis (for children under 2 years of age or when metabolic abnormalities are suspected).

#### Main outcomes

- Clinical assessment methods for short stature
- Diagnostic laboratory tests used for short stature evaluation
- Growth charts and reference standards used in diagnosis
- Diagnostic accuracy and test results interpretation
- Accuracy and application of target height formula in growth assessment

#### Study design

Only non-randomized study types will be included:

- Prospective and retrospective cohort studies
- Cross-sectional studies evaluating diagnostic accuracy
- Case-control studies comparing diagnostic methods
- Diagnostic accuracy studies
- Case series
- Systematic reviews and meta-analyses

#### Information sources

Systematic literature searches were conducted in PubMed, Embase, and Web of Science on 1^st^ July 2024, applying filters for English-language studies published between 2010 and 2024.

#### Search strategy

Literature search strategies were developed combining medical subject headings (MeSH) and free-text terms relevant to each domain. The full search strategy for the three databases is reported in Supplementary Table 1. for the clinical domain and Supplementary Table 2 for the biochemical domain

#### Selection process

The study selection process was conducted by two pairs of reviewers. LG and CLG independently screened studies for the clinical assessment domain, while CLG and GC independently screened studies for the biochemical assessment domain. Disagreements were resolved through discussion, with a third reviewer (CM) acting as an arbiter if needed. The study selection process was carried out in two phases: first, titles and abstracts were screened to assess adherence to inclusion criteria; second, reviewers analyzed the full texts of potentially eligible studies. Key characteristics of the included studies were defined ex ante and summarized in Supplementary tables, while a list of excluded studies, along with the reasons for exclusion, is presented in Supplementary Table 11 for the clinical domain and Supplementary Table 12 for the biochemical domain

#### Data collection process

Data extraction for both domains was conducted by pairs of reviewers (LG, CLG for the clinical domain; CLG and GC for the biochemical domain) using a standardized form. To ensure consistency across reviewers, calibration exercises were conducted before starting the review. Disagreements on data extracted were resolved through discussion, with a third reviewer (CM) acting as an arbiter if needed.

#### Data items

The following information was extracted: bibliographic data (first author, publication year), study characteristics (study design, study period, country, sample size), participant characteristics (gender, age at onset), outcome (epidemiological estimates), diagnostic criteria.

#### Risk of bias assessment

Included studies were assessed using the JBI Critical appraisal checklists for cross-sectional studies, cohort studies, diagnostic accuracy studies and case-series studies [8, 9]. The risk of bias assessment was performed by two independent reviewers (CLG, GC). Any discrepancies in judgements of risk of bias were resolved by discussion to reach consensus between the two reviewers, with a third reviewer (LG) acting as an arbiter if needed.

## Results

### Clinical assessment – selection process and data extraction

**Fig. 1:**
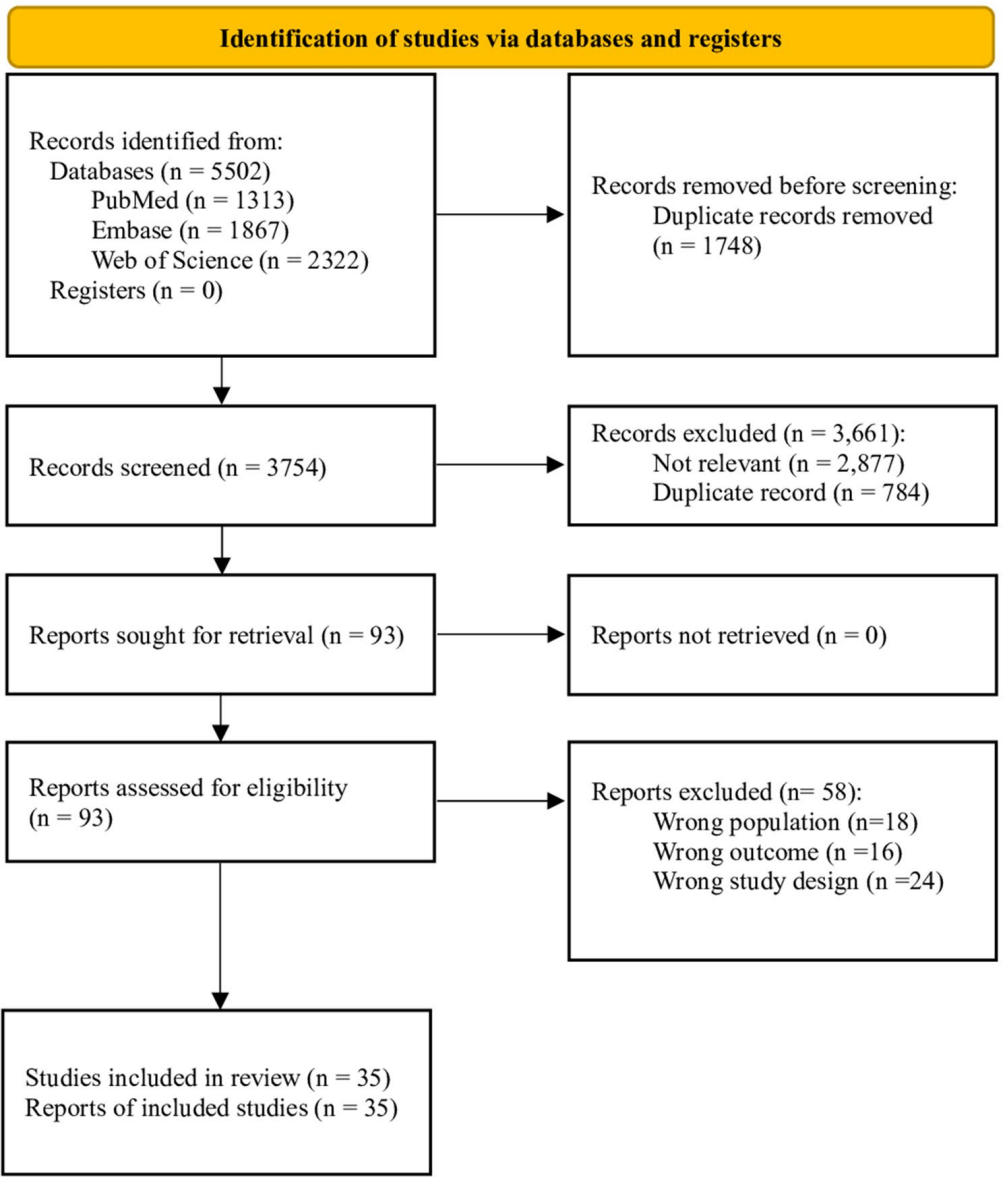
Clinical assessment selection process PRISMA 2020 flow diagram

The systematic literature review identified 5502 eligible records: 1313 from PubMed, 1867 from Embase and 2322 from Web of Science. After duplicates (*n* = 1748) removal, 3754 records were screened; of these, full texts of 93 were analyzed, resulting in the inclusion of 35 studies (Fig. 1).

Key characteristics of the included studies are summarized in Supplementary Table 3. Among the included studies, 19 were cross-sectional studies [10–28], 11 were population-based cohort studies [29], [30–34], [35–39], 1 was a diagnostic accuracy study [40], and 4 were case series [41–44]. A total of 18 studies were conducted in Asia, 9 in Europe, 4 in Africa, 3 in South America, and 1 in Canada.

Sample sizes ranged from a minimum of 65 individuals [20] to a maximum of 398,520 [17] with a median of 551 individuals. Thirty studies included both children and adolescents, 2 only adolescents [36, 40], and 3 studies only infants [14, 17, 34].

### Biochemical assessment - selection process and data extraction

**Fig. 2:**
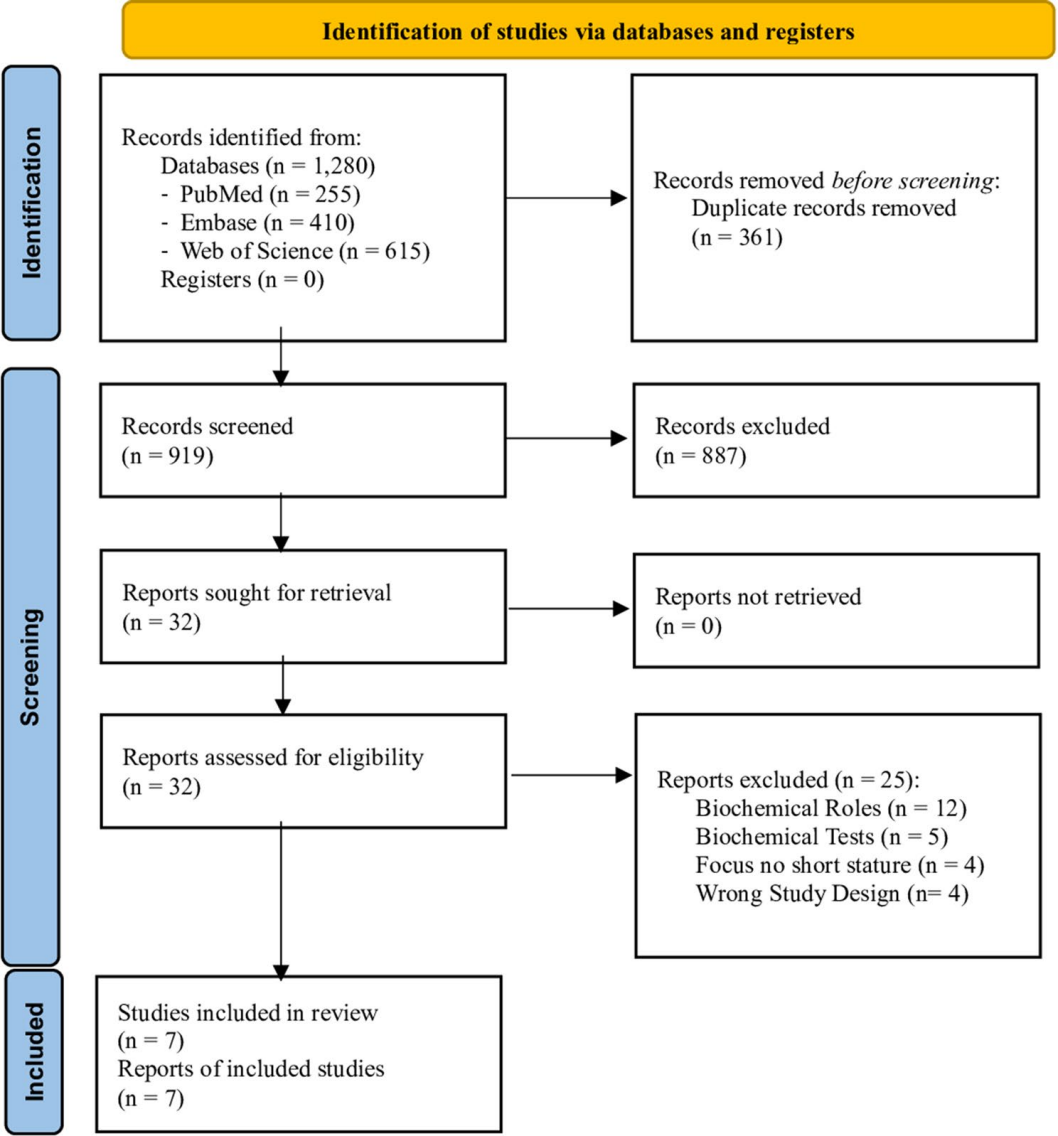
Biochemical assessment selection process PRISMA 2020 flow diagram

The systematic literature search identified 1280 eligible records: 255 from PubMed, 410 from Embase and 615 from Web of Science. After duplicates (*n* = 361) removal, 919 records were screened; of these, full texts of 32 were analyzed, resulting in the inclusion of 7 studies (Fig. 2).

Key characteristics of the included studies are summarized in Supplementary Table 4. Among the included studies, 2 were cross-sectional studies [45], [46], and 5 were cohort studies [47], [57] [48–50]. A total of 5 studies were conducted in Asia: 2 in Pakistan [46, 48], 2 in India [47, 49], and 1 in China [45]. One study was from Africa (Tunisia [51]), and 1 study was conducted in USA [50].

Sample sizes ranged from 45 [45] to 470 individuals [49], with a median of 145 individuals. All studies included children.

### Short stature definition

Regarding the definition of short stature, 28 out of the 42 included studies used the criterion of a height 2 Standard Deviation Score (SDS) below the average height for the individual’s age and sex, while 19 studies defined short stature as a height that falls below a percentile threshold on standardized growth chart: 17 using the 3^rd^ percentile and 2 using the 5^th^ percentile [21, 47], 3 studies do not report definition [10, 18]. [33].

A total of 8 studies used both criteria () to define short stature0.7 studies, meeting either the SDS-based criterion or the 3^rd^ percentile criterion was required, in 1 study [47] fulfilling the criterion based on SDS or the criterion based on the 5^th^ percentile was required.

### Reference standard charts

Studies using height locals national reference were [13, 17, 20, 26, 27, 30, 31, 36, 38, 43, 45]

When specific local/national chart was not available, international reference charts were employed: in these cases, the World Health Organization (WHO) ([10–12, 14–16, 18, 19, 22, 23, 25, 29, 39, 43]) and Centers for Disease Control and Prevention (CDC) growth charts ([13, 23, 24, 39, 41, 48]) were predominantly employed.

### Clinical examination tests: anthropometric measurements

International guidelines [6] recommend that the physical examination of a child referred for evaluation of short stature should include measurements of height, weight, BMI, head circumference, arm span, sitting height and sitting/standing height ratio.

Height was measured in all 35 included studies, while weight was assessed in 26 studies and BMI in 16. Head circumference measurements were included in 5 studies [14, 15, 17, 18, 36], arm span in 3 [28, 35, 36], and sitting/standing height ratio measurements were performed in 5 studies [18, 31, 33, 35, 36].

### Clinical examination tool and other auxological features

Bone age was assessed in 10 studies. In all studies, bone age was determined using the Greulich and Pyle method, except for Stalman et al. (2015) [35], which did not specify the method used.

Of the 15 studies that estimated target height, 9 employed the Tanner method (averaging the mother’s and father’s heights, adding 6.5 cm for boys and subtracting 6.5 cm for girls), 1 utilized the Tanner method with a correction for secular trends (adding 4.5 cm for both sexes after employing the Tanner method) [37], 1 calculated it by averaging the mother’s and father’s heights and then adding 6 cm for boys and subtracting 6 cm for girls [20] and 4 did not specify the method used [30, 31, 35, 36].

In the study by White et al. [37], height deflection, defined as the change in a child’s height SDS over time, was also evaluated.

### Biochemical tests

Complete blood count and urine and stool examination tests were conducted in all studies. Moreover, 6 studies [46–51] conducted TSH test; 5 studies [46–49, 51] performed thyroxine (T4) test; 2 studies [48, 50] included tissue transglutaminase (tTG) IgA antibodies and IgA tests; IGF-1 and IGFBP-3 levels were assessed in 2 studies [48, 50].

GH levels were evaluated in 3 studies [45, 47, 51]. In the study by Xu et al. [45], a biochemical analysis of serum was performed to assess indicators of liver function, renal function, blood lipids, and blood glucose.

### Risk of bias

Regarding the studies included in the clinical area, analysis of the 19 cross-sectional studies (Supplementary Table 5) indicated that 16 studies [10–17, 19, 20, 22–26, 28] met 80% or more of the 8 checklist items and 3 studies [18, 21, 27] met between 60 and 80% of the checklist items.

Among the 11 cohort studies (Supplementary Table 6) analyzed, 2 studies [33, 37] fulfilled 80% or more of the 11 checklist items, while 3 studies [30, 31, 34], met between 60 and 80%, 4 studies [4, 29, 35, 36] met between 40 and 60%, and 2 studies [32, 38] met less than 40% of the checklist items.

The analysis of the 4 case series studies (Supplementary Table 7) revealed that 3 studies [41–43] met more than 80% of the 10 checklist items, and 1 study [44] met between 60 and 80% of the checklist items.

Finally, the diagnostic accuracy study (Supplementary Table 8) [40] met 60% of the 10 checklist items.

Regarding the studies included in the biochemical area, among the 5 cohort studies (Supplementary Table 9) analyzed, 2 studies [47, 49]met between 60% and 80% of the 11 checklist items, and 3 studies [46, 48, 50] met between 40 and 60% of the checklist items.

The analysis of the 2 cross-sectional studies (Supplementary Table 10) [45, 46] indicated that the 2 studies fulfilled 80% or more of the 8 checklist items.

## Discussion

This systematic review analyzed diagnostic approaches to short stature in children, focusing on definitions, utilized reference growth charts, auxological measurements, and the laboratory tests employed in pediatric primary care.

Regarding the definition of short stature, our results showed a cut-off of an individual’s height < 2 SDS for the mean height of a given age, sex, and population group is the most used, followed by a height below the 3^rd^ percentile. Interestingly, despite height velocity provides a superior measure because changes in actual height only become evident after altered growth rates have been sustained for a period of time [52], this key auxological tool is rarely taken into consideration in most studies. Similarly, target height is rarely included when assessing a child with short stature.

Growth charts are an indispensable tool for monitoring the growth of children. Our results showed that local growth charts are mainly used to assess short stature when available, followed by WHO and CDC growth charts. Recently, the selection of the appropriate growth chart to be used as reference has become a subject of debate in many countries especially regarding the superiority of WHO growth chart compared to local ones [53, 54]. This debate is crucial because the proportion of children given a diagnosis of short stature varies significantly depending on the growth charts that are used as reference. Therefore, the use of the most appropriate growth chart will allow to avoid misdiagnosis.

Our results support the need to identify a definition of short stature universally agreed which ideally could take into consideration different factors which are known to influence child growth, including genetic variability, growth patterns, cultural and environmental factors. While there are general guidelines [3–6], such as considering children below the 3rd percentile or below − 2 SDS on growth charts as potentially having short stature, a standardized definition remains elusive, mainly based on expert opinions and not supported by the available studies published so far. Combining the subject’s height, its deviation from target height and height velocity could be more valuable than any isolated criterion in discriminating normal variant and pathological short stature.

Certainly, beside the need of well conducted studies in high income countries (not only in middle-low income ones) what is needed is more accurate information for general practitioners and physicians operating in that sector.

Approaching a child with short stature isn’t always straightforward, and clinicians often rely on individual growth assessments, family history, and additional diagnostic tests to determine if there is an underlying cause or if the child is simply experiencing a normal variation in growth.

Our results showed that complete blood count, urine and stool examination tests were performed in all studies as first-level examination tests. Interestingly, the TSH/FT4 test and screening for celiac disease were not universally performed when investigating a child with short stature. These findings reflect the absence of experimental evidence supporting the use of a universal laboratory screening program to detect a subclinical chronic illness that initially can present as growth failure. As suggested by Wit et al. the diagnostic yield of most components of this screening procedure is probably very low in children without any abnormality in the clinical evaluation and with a normal growth velocity [6], but on the other hand, the clinician would not like to miss a clinically relevant diagnosis. This situation has led to a wide variation among pediatric practices.

## Conclusion

This systematic review points out that significant variation among studies investigating children with short stature is present. The major critical issues concern the different definitions of short stature, the selection of growth charts, and the parameters used (e.g.: stature, growth velocity, target height) as well as different cut-off points (e.g., centiles vs. standard deviations). This variability could led to an increase in not-necessary specialist consultations, delay in more severe patients’ evaluation and increasing costs for public health system. Moreover worsening waiting time to receive care have been shown to be associated to patient dissatisfaction, and poorer clinical outcomes [55].

According to the available studies, different laboratory screening tests are used when approaching a child with short stature.

## Electronic supplementary material

Below is the link to the electronic supplementary material.

Supplementary Material 1

## Data Availability

Materials about this study can be obtained from the corresponding author on reasonable request.

## Abbreviations

BMI: Body Mass Index
CBC: Cello blood count
CDC: Centers for Disease Control and Prevention
HV: Height velocity
SDS: Standard deviation score
WHO: World Health Organization
