# Supplementary material for: Clinical and biochemical evaluation of children with short stature in the primary care setting: a systematic review
Source: Ital J Pediatr. 2026 Feb 21;52:47. doi: 10.1186/s13052-026-02215-8 (PMC13032349; doi:10.1186/s13052-026-02215-8)
Supplement: Supplementary file 1 — Supplementary Material 1 [file 13052_2026_2215_MOESM1_ESM.docx]

| Table 1. Search strategy – Clinical area | | |
| --- | --- | --- |
| **PubMed, 01/07/2024** | | |
| Search | Query | Results |
| #1 | (short stature [Title/Abstract] OR stunted growth [Title/Abstract] OR growth delay [Title/Abstract] OR short child* [Title/Abstract]) | 19,969 |
| #2 | (child OR children OR pediatric OR pediatrics OR paediatric OR paediatrics OR infant OR infants OR infancy OR newborn OR newborns OR adolescent OR adolescents OR juvenile OR youth OR toddler OR toddlers OR kid OR kids OR boy OR boys OR girl OR girls OR baby OR babies OR teen OR teens OR preteen OR preteens OR teenager OR teenagers OR pubescen* OR prepubescen* OR neonate OR neonates OR (allchild[Filter] OR newborn[Filter] OR allinfant[Filter] OR infant[Filter] OR child[Filter] OR adolescent[Filter] OR preschoolchild[Filter])) | 6,846,994 |
| #3 | diagnosis[Title/Abstract] OR diagnostic[Title/Abstract] OR ((clinical[Title/Abstract] OR medical[Title/Abstract] OR physical[Title/Abstract]) AND (assessment[Title/Abstract] OR evaluation[Title/Abstract] OR examination[Title/Abstract])) OR ((growth[Title/Abstract] OR height[Title/Abstract] OR weight[Title/Abstract]) AND (assessment[Title/Abstract] OR evaluation[Title/Abstract] OR monitoring[Title/Abstract])) | 3,716,062 |
| #4 | #1 AND #2 AND #3 | 4,743 |
| #5 | #4 NOT ("case reports"[Publication Type] OR "case report"[Title] OR "case reports"[Title] OR "editorial"[Publication Type] OR "letter"[Publication Type] OR "comment"[Publication Type]) | 3,177 |
| #6 | #5 AND (humans[Filter]) AND (english[Filter]) AND ("2010"[Date - Publication] : "3000"[Date - Publication]) | 1,313 |
| **Embase, 01/07/2024** | | |
| Search | Query | Results |
| #1 | ('short stature':ti,ab,kw OR 'stunted growth':ti,ab,kw OR 'growth delay':ti,ab,kw OR 'short child*':ti,ab,kw) | 30,031 |
| #2 | child:ti,ab,kw OR children:ti,ab,kw OR pediatric:ti,ab,kw OR pediatrics:ti,ab,kw OR paediatric:ti,ab,kw OR paediatrics:ti,ab,kw OR infant:ti,ab,kw OR infants:ti,ab,kw OR infancy:ti,ab,kw OR newborn:ti,ab,kw OR newborns:ti,ab,kw OR adolescent:ti,ab,kw OR adolescents:ti,ab,kw OR juvenile:ti,ab,kw OR youth:ti,ab,kw OR toddler:ti,ab,kw OR toddlers:ti,ab,kw OR kid:ti,ab,kw OR kids:ti,ab,kw OR boy:ti,ab,kw OR boys:ti,ab,kw OR girl:ti,ab,kw OR girls:ti,ab,kw OR baby:ti,ab,kw OR babies:ti,ab,kw OR teen:ti,ab,kw OR teens:ti,ab,kw OR preteen:ti,ab,kw OR preteens:ti,ab,kw OR teenager:ti,ab,kw OR teenagers:ti,ab,kw OR pubescen*:ti,ab,kw OR prepubescen*:ti,ab,kw OR neonate:ti,ab,kw OR neonates:ti,ab,kw | 3,557,363 |
| #3 | diagnosis:ti,ab,kw OR diagnostic:ti,ab,kw OR ((clinical:ti,ab,kw OR medical:ti,ab,kw OR physical:ti,ab,kw) AND (assessment:ti,ab,kw OR evaluation:ti,ab,kw OR examination:ti,ab,kw)) OR ((growth:ti,ab,kw OR height:ti,ab,kw OR weight:ti,ab,kw) AND (assessment:ti,ab,kw OR evaluation:ti,ab,kw OR monitoring:ti,ab,kw)) | 5,457,431 |
| #4 | #1 AND #2 AND #3 | 6,960 |
| #5 | #4 AND ([article]/lim OR [article in press]/lim OR [review]/lim) AND [english]/lim AND [humans]/lim AND [2010-2024]/py | 2,090 |
| #6 | #5 NOT 'case report*':ti | 1,867 |
| **Web of Science, 01/07/2024** | | |
| Search | Query | Results |
| #1 | TS=("short stature" OR "stunted growth" OR "growth delay" OR "short child*") | 21,289 |
| #2 | TS=(child OR children OR pediatric OR pediatrics OR paediatric OR paediatrics OR infant OR infants OR infancy OR newborn OR newborns OR adolescent OR adolescents OR juvenile OR youth OR toddler OR toddlers OR kid OR kids OR boy OR boys OR girl OR girls OR baby OR babies OR teen OR teens OR preteen OR preteens OR teenager OR teenagers OR pubescen* OR prepubescen* OR neonate OR neonates) | 3,395,140 |
| #3 | TS=(diagnosis OR diagnostic OR ((clinical OR medical OR physical) AND (assessment OR evaluation OR examination)) OR ((growth OR height OR weight) AND (assessment OR evaluation OR monitoring))) | 4,595,805 |
| #4 | #1 AND #2 AND #3 | 4,140 |
| #5 | #1 AND #2 AND #3 and Article or Review Article (Document Types) and English (Languages) and 2024 or 2023 or 2022 or 2021 or 2020 or 2019 or 2018 or 2017 or 2016 or 2015 or 2014 or 2013 or 2012 or 2011 or 2010 (Publication Years) | 2,544 |
| #6 | #5 NOT TI=("case report*" OR letter OR editorial OR comment) | 2,322 |

| Table 2. Search strategy – Biochemical area | | |
| --- | --- | --- |
| **PubMed, 01/07/2024** | | |
| Search | Query | Results |
| #1 | (short stature [Title/Abstract] OR stunted growth [Title/Abstract] OR growth delay [Title/Abstract] OR short child* [Title/Abstract]) | 19,969 |
| #2 | (child OR children OR pediatric OR pediatrics OR paediatric OR paediatrics OR infant OR infants OR infancy OR newborn OR newborns OR adolescent OR adolescents OR juvenile OR youth OR toddler OR toddlers OR kid OR kids OR boy OR boys OR girl OR girls OR baby OR babies OR teen OR teens OR preteen OR preteens OR teenager OR teenagers OR pubescen* OR prepubescen* OR neonate OR neonates OR (allchild[Filter] OR newborn[Filter] OR allinfant[Filter] OR infant[Filter] OR child[Filter] OR adolescent[Filter] OR preschoolchild[Filter])) | 6,846,994 |
| #3 | biochemical [Title/Abstract] AND (evaluation* [Title/Abstract] OR diagnosis [Title/Abstract] OR marker* [Title/Abstract] OR investigation* [Title/Abstract] OR screening* [Title/Abstract] OR test* [Title/Abstract] OR exam*[Title/Abstract]) | 197,965 |
| #4 | biochemistry [Title/Abstract] OR "celiac disease" [Title/Abstract] OR Intestinal chronic disease [Title/Abstract] OR "inflammatory disorder*" [Title/Abstract] OR "hepatic function alteration*" [Title/Abstract] OR "kidney function alteration*" [Title/Abstract] OR "chronic cardiopathy" [Title/Abstract] OR "thyroid function" [Title/Abstract] hypothyroidism [Title/Abstract] OR "karyotype alteration*" [Title/Abstract] OR "chromosomal alteration*" [Title/Abstract] OR calcium-phosphorus [Title/Abstract] OR "differential diagnosis" [Title/Abstract] OR "hormon* exam*"[Title/Abstract] | 157,437 |
| #5 | (test [Title/Abstract] OR assay [Title/Abstract]) AND ("gh stimulation"[Title/Abstract] OR igfbp3[Title/Abstract] OR "insulin tolerance"[Title/Abstract] OR "glucagon stimulation"[Title/Abstract] OR "gh-releasing peptide"[Title/Abstract] OR clonidine[Title/Abstract] OR l-dopa[Title/Abstract] OR arginine[Title/Abstract] OR ghrh-arginine[Title/Abstract] OR "insulin-like growth factor 1" [Title/Abstract] OR igf1 [Title/Abstract] OR somatomedin C[Title/Abstract]) | 20,824 |
| #6 | #3 OR #4 OR #5 | 372,587 |
| #7 | #1 AND #2 AND #6 | 1,081 |
| #8 | #7 NOT ("case reports"[Publication Type] OR "case report"[Title] OR "case reports"[Title] OR "editorial"[Publication Type] OR "letter"[Publication Type] OR "comment"[Publication Type]) | 750 |
| #9 | #8 AND (humans[Filter]) AND (english[Filter]) AND ("2010"[Date - Publication] : "3000"[Date - Publication]) | 255 |
| **Embase, 01/07/2024** | | |
| Search | Query | Results |
| #1 | ('short stature':ti,ab,kw OR 'stunted growth':ti,ab,kw OR 'growth delay':ti,ab,kw OR 'short child*':ti,ab,kw) | 30,031 |
| #2 | child:ti,ab,kw OR children:ti,ab,kw OR pediatric:ti,ab,kw OR pediatrics:ti,ab,kw OR paediatric:ti,ab,kw OR paediatrics:ti,ab,kw OR infant:ti,ab,kw OR infants:ti,ab,kw OR infancy:ti,ab,kw OR newborn:ti,ab,kw OR newborns:ti,ab,kw OR adolescent:ti,ab,kw OR adolescents:ti,ab,kw OR juvenile:ti,ab,kw OR youth:ti,ab,kw OR toddler:ti,ab,kw OR toddlers:ti,ab,kw OR kid:ti,ab,kw OR kids:ti,ab,kw OR boy:ti,ab,kw OR boys:ti,ab,kw OR girl:ti,ab,kw OR girls:ti,ab,kw OR baby:ti,ab,kw OR babies:ti,ab,kw OR teen:ti,ab,kw OR teens:ti,ab,kw OR preteen:ti,ab,kw OR preteens:ti,ab,kw OR teenager:ti,ab,kw OR teenagers:ti,ab,kw OR pubescen*:ti,ab,kw OR prepubescen*:ti,ab,kw OR neonate:ti,ab,kw OR neonates:ti,ab,kw | 3,557,363 |
| #3 | biochemical:ti,ab,kw AND (evaluation*:ti,ab,kw OR diagnosis:ti,ab,kw OR marker*:ti,ab,kw OR investigation*:ti,ab,kw OR screening*:ti,ab,kw OR test*:ti,ab,kw OR exam*:ti,ab,kw) | 287,283 |
| #4 | biochemistry:ti,ab,kw OR 'celiac disease':ti,ab,kw OR 'intestinal chronic disease':ti,ab,kw OR 'inflammatory disorder*':ti,ab,kw OR 'hepatic function alteration*':ti,ab,kw OR 'kidney function alteration*':ti,ab,kw OR 'chronic cardiopathy':ti,ab,kw OR 'thyroid function':ti,ab,kw hypothyroidism:ti,ab,kw OR 'karyotype alteration*':ti,ab,kw OR 'chromosomal alteration*':ti,ab,kw OR 'calcium-phosphorus':ti,ab,kw OR 'differential diagnosis':ti,ab,kw OR 'hormon* exam*':ti,ab,kw | 223,374 |
| #5 | (test:ti,ab,kw OR assay:ti,ab,kw) AND ('gh stimulation':ti,ab,kw OR igfbp3:ti,ab,kw OR 'insulin tolerance':ti,ab,kw OR 'glucagon stimulation':ti,ab,kw OR 'gh-releasing peptide':ti,ab,kw OR clonidine:ti,ab,kw OR 'l-dopa':ti,ab,kw OR arginine:ti,ab,kw OR 'ghrh-arginine':ti,ab,kw OR 'insulin-like growth factor 1':ti,ab,kw OR igf1:ti,ab,kw OR 'somatomedin C':ti,ab,kw) | 30,689 |
| #6 | #3 OR #4 OR #5 | 535,068 |
| #7 | #1 AND #2 AND #6 | 1,741 |
| #8 | #7 AND ([article]/lim OR [article in press]/lim OR [review]/lim) AND [english]/lim AND [humans]/lim AND [2010-2024]/py | 448 |
| #9 | #8 NOT 'case report*':ti | 410 |
| **Web of Science, 01/07/2024** | | |
| Search | Query | Results |
| #1 | TS=("short stature" OR "stunted growth" OR "growth delay" OR "short child*") | 21,289 |
| #2 | TS=(child OR children OR pediatric OR pediatrics OR paediatric OR paediatrics OR infant OR infants OR infancy OR newborn OR newborns OR adolescent OR adolescents OR juvenile OR youth OR toddler OR toddlers OR kid OR kids OR boy OR boys OR girl OR girls OR baby OR babies OR teen OR teens OR preteen OR preteens OR teenager OR teenagers OR pubescen* OR prepubescen* OR neonate OR neonates) | 3,395,140 |
| #3 | TS=(biochemical AND (evaluation* OR diagnosis OR marker* OR investigation* OR screening* OR test* OR exam*)) | 229,843 |
| #4 | TS=(biochemistry OR “celiac disease” OR intestinal chronic disease OR “inflammatory disorder*” OR “hepatic function alteration*” OR “kidney function alteration*” OR “chronic cardiopathy” OR “thyroid function” hypothyroidism OR “karyotype alteration*” OR “chromosomal alteration*” OR calcium-phosphorus OR “differential diagnosis” OR “hormon* exam*”) | 252,048 |
| #5 | TS=((test OR assay) AND (“gh stimulation” OR igfbp3 OR “insulin tolerance” OR “glucagon stimulation” OR “gh-releasing peptide” OR clonidine OR l-dopa OR arginine OR ghrh-arginine OR “insulin-like growth factor 1” OR igf1 OR “somatomedin C”)) | 39,910 |
| #6 | #3 OR #4 OR #5 | 511,435 |
| #7 | #1 AND #2 AND #6 | 1,272 |
| #8 | #1 AND #2 AND #6 and Article or Review Article (Document Types) and English (Languages) and 2024 or 2023 or 2022 or 2021 or 2020 or 2019 or 2018 or 2017 or 2016 or 2015 or 2014 or 2013 or 2012 or 2011 or 2010 (Publication Years) | 660 |
| #9 | #8 NOT TI=("case report*" OR letter OR editorial OR comment) | 615 |

| Table 3. Key characteristics of included studies – Clinical domain | | | | | | | |
| --- | --- | --- | --- | --- | --- | --- | --- |
| **Study ID**  **[Reference]** | **Country** | **Study Design** | **Population (age, sex)** | **Sample Size** | **Definition of short stature** | **Clinical examination tests** | **Reference standard charts** |
| Ahmad 2021  [10] | Pakistan | Cross-sectional study | Children aged 2-12 years old; M 42%; (mean age ± SD: 4.81 ±2.66); F 58% (mean age ± SD: 5.33 ±3.13) | 124 | Not reported | Height, weight and BMI | NCHS/WHO growth standards |
| Aizpurua Galdeano 2019 [11] | Spain | Population based cross-sectional study | Children turned 4, 6, 10 or 13 years of age in 2015;  M 51%, F 49% | 12256 | Height below the 3rd percentile | Height | FO04 L and FO11 charts (primary objective) FO88, ETE10, FO04 CS growth charts (secondary objective) and WHO growth standards |
| Al-Abdulrazzaq 2016 [12] | Kuwait | Cross-sectional study | Children and adolescents aged 0 -18 years;  Median age (IQR): 7.7 (4.7-10.3 years);  M 53%, F 47% | 221 | Height below the 3rd percentile or height <–2 standard deviation score (SDS) | Height, weight, BMI, mid-parental target height and pubertal status (Tanner stages) | WHO growth standards |
| Alassaf 2021 [13] | Jordan | Retrospective cohort study | Children and adolescents aged 0 -18 years;  Mean age (SD): 10.24 (3.23) years;  M 55%, F 45% | 551 | Height <–2 standard deviation score (SDS) | Height, BMI, mid-parental target height, bone age (Greulich–Pyle method) and pubertal status (Tanner stages) | WHO growth standards |
| Aroor 2014 [14] | India | Cross-sectional study | Children and adolescents aged 4-16 years;  mean age not reported;  M 48%, F 52% | 755 | Height below the 3rd percentile | Height, weight and body mass index (BMI) | CDC and Agarwal growth charts |
| Bouferoua 2023 [15] | Algeria, UK | Prospective cohort study | Neonates with gestational age between 37 and 42 weeks, from a single pregnancy and with a birth weight and/or length below the 10th percentile (SGA);  M 44%, F 56% | 457 | Height <–2 standard deviation score (SDS) | Height, weight, head circumference, mid-parental target height and catch-up growth | WHO growth standards |
| Buhendwa 2017 [16] | Democratic Republic of Congo, Belgium | Cross-sectional study | Children and adolescents aged 6-18 years;  mean age not reported;  M 48%, F 52% | 7541 | Height <–2 standard deviation score (SDS) | Height, weight, BMI and head circumference | WHO growth standards |
| Bundak 2022 [17] | Turkey | Population based cross-sectional study | Children and adolescents aged 0-18 years;  mean age not reported;  M 53%, F 47% | 6613 | Height below the 3rd percentile | Height, weight and BMI | WHO growth standards |
| Cha 2025 [18] | Korea | Population based cross-sectional study | Newborns with follow-up until 6 years of age;  M 52%; F 48% (preterm);  M 41%; F 59% (full term low birth weight);  M 47%, F 53% (controls) | 41454 PT; 38250 FT-LBW; 318816 controls | Height <–2 standard deviation score (SDS) | Height, weight and head circumference | Korean National Growth Charts for Children and Adolescents (KNGC2017) |
| Del Pino 2017 [19] | Argentina | Cross-Sectional study | Children and adolescents aged 0-17 years;  mean age not reported;  M 49.9%, F 50.1% | 9621 | Not reported | Sitting height/height (SH/H) Head circumference/height (HC/H) | WHO growth standards |
| El-Shafie 2020 [20] | Egypt | Cross-Sectional study | Children aged  6-11 years;  mean age not reported;  M 51.7%, F 48.3% | 33150 | Height <–2 standard deviation score (SDS) | Height, weight, BMI, mid-parental target height, bone age (Greulich–Pyle method) and pubertal status (Tanner stages) | WHO growth standards |
| Grigoletto 2021 [21] | Italy | Cross-Sectional study | Children;  Median age (IQR)  9.1 (6.2;12.4); M 58.5%, F 41.5% | 65 | Height <–2 standard deviation score (SDS) | Height, weight, BMI, mid-parental height and pubertal status (Tanner stages) | Italian reference charts (Cacciari 2006) |
| Kandagal 2023 [22] | India | Prospective Observational study | Children aged 6-11 years;  mean age not reported | 1128 | Height <–2 standard deviation score (SDS) | Height, weight, BMI, mid-parental target height and bone age (Greulich–Pyle method) | Indian Academy of Pediatrics (IAP) growth charts |
| Karkinen 2020 [23] | Finland | Retrospective study | Children;  Mean age (SD):  9.2 (5.2) years; M 52.1%, F 47.9% | 785 | Height <–2 standard deviation score (SDS) | Height, target height, sitting height/height ratio | Finnish and Dutch reference data |
| Lev Ran 2013 [24] | Israel | Cross-sectional study | Children;  mean age not reported;  M 50.3%, F 49.7% | 24846 | Height below the 5th percentile | Height, weight, standard physical examination | Not reported |
| Lucio 2019 [25] | Brazil | Diagnostic accuracy study | Adolescents aged 10 - 19 years | 385 | Height below the 3rd percentile | Delayed sexual maturation, decreased bone mass for age and sex, stature below genetic target, growth velocity (GV) less than expected, low weight for age and sex and short stature for age and sex | Not reported |
| Ma 2019 [26] | China | Survey | Children and adolescents aged 7-18 years M 49.98%, F 50.02% | 213795 | Height below the 3rd percentile | Height | 2006 WHO  growth standards and Chinese growth reference |
| Machado 2012 [27] | Brazil | Case series | Children aged 5-10 years; M 54.5%, F 45.5% | 157 | -2 Z scores | Height, weight, BMI | CDC growth charts, 2000 |
| Markosyan 2014 [28] | Armenia | Cohort study | Children and adolescents aged 5.8-18.5 years; Mean age (SD):  10.8 (3.3) years M 53.5%, F 45.5% | 1500 | Height <–2 standard deviation score (SDS) | Height | Not reported |
| Moelyo 2022 [29] | Indonesia | Cross-sectional | Children and adolescents aged 6-18.5 years; Mean age (SD):  13.1 (3.4) years M 36.9%, F 63.1% | 2582 | Height <–2 standard deviation score (SDS) | Height, weight, and BMI | WHO Growth Chart for children aged 5-19 years,4 the CDC 2000 Growth Chart,8 and the National Indonesian Growth Chart (NIGC). |
| Muñoz 2013 [30] | Spain | Longitudinal study | Children and adolescents aged 0-18 years; M 49.7%, F 50.3% | 332 | Not reported | Sitting/standing height ratio | Not reported |
| Papadimitriou 2012 [31] | Greece | Case series | Children M 54.9%, F 45.1% | 295 | Height below the 3rd percentile or height <–2 standard deviation score (SDS) | Height, weight, BMI, mid-parental target height, bone age (Greulich–Pyle method), height velocity, Height velocity standard deviation score | Not reported |
| Prasad 2012 [32] | India | Case series | Children aged 0-5 years;  Mean age (SD): 2.8 (1.2) years | 1840 | Height below the 3rd percentile | Height and weight | WHO growth standards, Indian Academy of Pediatrics references |
| Rabbani 2013 [33] | Pakistan | Cross-sectional | Children and adolescents aged 2-15 years; M 53.8%, F 46.2% | 169 | Height below the 3rd percentile or height <–2 standard deviation score (SDS) | Height, weight, mid-parental target height, upper to lower segment ratio, bone age (Greulich–Pyle method) and pubertal status (Tanner stages) | 2000 CDC growth charts |
| Ramadan 2021 [34] | Libya | Cross-sectional | Children and adolescents aged 6-15.4 years;  Mean age (SD):  10.67 (2.6); M 56.1%, F 43.9% | 419 | Height <–2 standard deviation score (SDS) | Height, weight and BMI | WHO 2007‑Z Score growth charts; UK‑WHO growth charts |
| Saengkaew 2017 [35] | Thailand | Retrospective observational study | Children and adolescents aged 0-20 years; M 60,5%, F 39,5% | 521 | Severe short children: Height <-3 SDS;  moderate short stature: height between −2 SDS and −3 SDS | Height, weight, BMI, mid-parental target height, bone age (Greulich–Pyle method) and pubertal status (Tanner stages) | Not reported |
| Sardar 2015 [36] | UK | Cohort study | Live births M 51%, F 49% | 3798;  107 met the criteria for follow-up | Height <–2 standard deviation score (SDS) | Height, weight, mid-parental height, catch-up growth | Not reported |
| Sharma 2023 [37] | India | Cross-sectional | Children and adolescents aged 5-16 years; Mean (SD) age: 11.7 (2.6); M 46.8%, F 53.2% | 4189 | Height below the 3rd percentile | Height | Indian Academy of Pediatrics (IAP) Khalidkar’s growth chart |
| Stalman 2015 [38] | The Netherlands | Cohort study | Children aged 3-10 years; M 58.0%, F 42.0% | 131 | Height <–2 standard deviation score (SDS) | All patients underwent a standardized diagnostic workup which includes an evaluation of the medical and family history, detailed auxological measurements (including height, weight, arm span, mid-parental target height, sitting height/height ratio), bone age assessment and a physical examination with special attention to dysmorphisms and body disproportions. | ESPE Classification |
| Stalman 2016 [39] | The Netherlands | Cohort study | Adolescents aged 10-18 years; M 54.4%, F 45.6% | 182 | Height <–2 standard deviation score (SDS) | Height, weight, head circumference, sitting height/height ratio, arm span, mid-parental target height, bone age (Greulich–Pyle method) and pubertal status (Tanner stages) and growth history | Dutch reference data |
| Takaya 2022 [40] | Japan | Cross-sectional | Schoolchildren and adolescents | 139435 | Height <–2 standard deviation score (SDS) | Height and weight | Data released by the Ministry of Health, Labour and Welfare and the Ministry of Education, Culture, Sports, Science and Technology of Japan in 2000 |
| Ullah 2016 [41] | Pakistan | Cross-sectional | Children and adolescents aged 2-20 years; Mean (SD) age: 11.75 (4.06); M 56.2%, F 43.8 | 73 | Height below the 3rd percentile or height <–2 standard deviation score (SDS) | Standing height, sitting height, arm span, lower and upper body segments heights, weight, mid-parental target height, bone age (Greulich–Pyle method) and pubertal status (Tanner stages) and growth history | Tanner and Davies growth chart |
| White 2022 [42] | UK | Case series | Children and adolescents aged 0.5 – 19.9 years; Mean age: 8.7 M 72%, F 28% | 143 | Height <–2 standard deviation score (SDS) (in line with Dutch definition) | Height, mid-parental target height, height deflection | UK and Dutch growth referral criteria |
| Yang 2010 [43] | Taiwan | Retrospective study | Children (age not reported); M 41%, F 59% | 139 | Height below the 3rd percentile | Height, weight and bone age (Greulich–Pyle method) | Growth charts of Taiwanese youth |
| Yue 2019 [44] | Canada | Retrospective chart review | Children and adolescents from 11 months to 18 years; Mean (SD) age: 9.5 (4.4); M 67.5%, F 32.5% | 286 | Height <–2 standard deviation score (SDS) | Height, weight, height velocity and mid-parental target height. | WHO growth charts and CDC growth charts |
| **Abbreviations: BMI,** Body Mass Index**; CDC,** Centers for Disease Control and Prevention**; ESPE,** European Society for Paediatric Endocrinology**; ETE10, “**Estudio Transversal Espanol 2010”**; FO04 CS, “**Fundaction Orbegozo Charts of 2004”**; FO11, “**Fundaction Faustino Orbegozo 2011”**; FO88, “**Fundaction Faustino Orbegozo 1988”**; FT,** Full-Term**; GV,** Growth Velocity**; H,** Height**; HC,** Head Circumference**; IAP,** Indian Academy of Pediatrics**; IQR,** Inter-Quartile Range**; KNGC2017,** Korean National Growth Charts 2017**; LBW,** Low Birth Weight**; NCHS,** National Center for Health Statistics**; NIGC,** National Indonesian Growth Chart**; PT,** Pre-Term**; SD,** Standard Deviation**; SDS,** Standard Deviations Score**; SH,** Sitting Height**; UK,** United Kingdom **WHO,** Word Health Organization | | | | | | | |

| Table 4. Key characteristics of included studies – Biochemical domain | | | | | | | |
| --- | --- | --- | --- | --- | --- | --- | --- |
| **Study ID**  **[Reference]** | **Country** | **Study Design** | **Population (age, sex)** | **Sample Size** | **Definition of short stature** | **Biochemical Screening Test** | **Reference standard charts** |
| Bhadada 2010 [46] | India | Retrospective cohort study | Children range: 10-15 Years  1)1995-1996; M 54% F 46%  2) 2005-2007; 54% F 46%" | 1) 1995-1996; 190  2)2005-2007; 256 | Height <–2 standard deviation score (SDS) or height below the 5th percentile | Complete blood count, Urine and stool examination, Biochemical tests, TSH (Thyroid-stimulating hormone) Test, Thyroxine (T4) Test | NCHS growth charts |
| Essaddam 2019 [47] | Tunisia | Retrospective study | Children Range: 0.5 – 17.5 Years M 57% F 43% | 470 | Height <–2 standard deviation score (SDS) or height below the 3rd percentile | Blood count, free T4, thyroidstimulating hormone (TSH), transglutaminase antibodies | Sempe and Pedron growth charts |
| Jawa 2016 [48] | Pakistan | Retrospective study | Children Range: 2 - 18 Years M 54% F 46% | 70 | Height <–2 standard deviation score (SDS) or height below the 3rd percentile | Complete blood count, fasting serum calcium, phosphorus and alkaline phosphatase and urinary pH, TSH test, T4 | CDC growth charts |
| Lashari 2014 [49] | Pakistan | Cross-sectional study | Children Range: 3-15 Years M 48% F 52% | 100 | Height <–2 standard deviation score (SDS) | Complete blood count, ESR, Ca, P, Alk - P, TSH test, T4, T3 | National Centre for Health Statistics (NCHS) |
| Mali 2013 [50] | India | Prospective study | Children Range: 0-15 Years M 57% F 43% | 100 | Height below the 3rd percentile | Blood test, Thyroid stimulating hormone (TSH) test, Thyroxine (T4) Test, Anti tTG | National Centre for Health Statistics (NCHS) |
| Sisley 2013 [51] | USA | Retrospective cohort study | Children Range: 0,8-17,6 Years M 72.8% F 28.2% | 235 | Height below the 3rd percentile | Complete blood count, Basic metabolic panel, Calcium, Phosphorus, Alkaline phosphatase, tTG, IgA, IGF 1, IGFBP3 , TSH, Thyroxine (T4) test | Not reported |
| Xu 2019 [45] | China | Observational study | Children Range: 5-15 Years M 57% F 43% | 45 | Height <–2 standard deviation score (SDS) or height below the 3rd percentile | Clinical biochemical analyses of serum were performed on an automatic biochemical analyzer (Mairui BS-350E, China). A total of 19 biochemical indicators falls into four categories: liver function, renal function, blood lipid, and blood glucose | Reference table of height and weight of Chinese children |
| **Abbreviations: Alk-P, A**lkaline Phosphatase**; Ca,** Calcium**; CDC, C**entre for Disease Control and Prevention**; ESR,** Erythrocyte Sedimentation Rate**; FT4,** Free Thyroxine**; GH,** Growth Hormone**; GHT,** Growth Hormone Test**; IGFBP-3**, Insulin-Like Growth Factor-Binding Protein**; IGF-I,** Insulin.Like Growth Factor 1**; MPH,** Mid Parental Target Height; **NCHS**, National Centre for Health Statistics; **P**, Phosphorus; **SD,** Standard Deviation; **SDS,** Standard Deviation Score; **T3**, Triiodothyronine; **T4**, Thyroxine Test; **TSH**, Thyroid-Stimulating Hormon Test; **tTG**, Anti-Tissue Transglutaminase; **WHO**, Word Health Organization. | | | | | | | |

| Table 5. Cross-sectional studies quality assessment (JBI checklist) – Clinical area | | | | | | | | |
| --- | --- | --- | --- | --- | --- | --- | --- | --- |
| Study | Item 1 | Item 2 | Item 3 | Item 4 | Item 5 | Item 6 | Item 7 | Item 8 |
| Ahmad 2021 [10] | Yes | Yes | Yes | Yes | Yes | Yes | Yes | Yes |
| Aizpurua Galdeano 2019 [11] | Yes | Yes | Yes | Yes | Yes | Yes | Yes | Yes |
| Al-Abdulrazzaq 2016 [12] | Yes | Yes | Yes | Yes | Yes | Yes | Yes | Yes |
| Aroor 2014 [14] | Yes | Yes | Yes | Yes | Yes | Yes | Yes | Yes |
| Bouferoua 2023 [15] | Yes | Yes | Yes | Yes | Yes | Yes | Yes | Yes |
| Buhendwa 2017 [16] | Yes | Yes | Yes | Yes | Yes | Yes | Yes | Yes |
| Bundak 2022 [17] | Yes | Yes | Yes | Yes | Yes | Yes | Yes | Yes |
| Cha 2025 [18] | Yes | Yes | Yes | Yes | Yes | Yes | Yes | Yes |
| Del Pino 2017 [19] | Yes | Yes | Yes | Yes | No | No | Yes | Yes |
| El-Shafie 2020 [20] | Yes | Yes | Yes | Yes | Yes | Yes | Yes | Yes |
| Grigoletto 2021 [21] | Yes | Yes | Yes | Yes | Yes | Yes | Yes | Yes |
| Lev Ran 2013 [24] | Yes | Yes | Yes | Yes | No | No | Yes | Yes |
| Ma 2019 [26] | Yes | Yes | Yes | Yes | Yes | Yes | Yes | Yes |
| Moelyo 2022 [29] | Yes | Yes | Yes | Yes | Yes | Unclear | Yes | Yes |
| Rabbani 2013 [33] | Yes | Yes | Yes | Yes | Yes | Yes | Yes | Yes |
| Ramadan 2021 [34] | Yes | Yes | Yes | Yes | Yes | Yes | Yes | Yes |
| Sharma 2023 [37] | Yes | Yes | Yes | Yes | Yes | Yes | Yes | Yes |
| Takaya 2022 [40] | Yes | Yes | Yes | Yes | No | No | Yes | Yes |
| Ullah 2016 [41] | Yes | Yes | Yes | Yes | Yes | Yes | Yes | No |
| Items   1. Were the criteria for inclusion in the sample clearly defined? 2. Were the study subjects and the setting described in detail? 3. Was the exposure measured in a valid and reliable way? 4. Were objective standard criteria used for measurement of the condition? 5. Were confounding factors identified? 6. Were strategies to deal with confounding factors stated? 7. Were the outcomes measured in a valid and reliable way? 8. Was appropriate statistical analysis used? | | | | | | | | |

| Table 6. Cohort studies quality assessment (JBI checklist) – Clinical area | | | | | | | | | | |
| --- | --- | --- | --- | --- | --- | --- | --- | --- | --- | --- |
| Study | Item 1 | Item 2 | Item 3 | Item 4 | Item 5 | Item 6 | Item 7 | Item 8 | Item 9 | Item 10 |
| Alassaf 2021 [13] | Not Applicable | Not Applicable | Yes | Yes | Yes | Yes | Yes | Not Applicable | Not Applicable | Not Applicable |
| Kandagal 2023 [22] | Not Applicable | Not Applicable | Yes | Unclear | Unclear | Yes | Yes | Yes | Yes | Yes |
| Karkinen 2020 [23] | Yes | Yes | Yes | Yes | Yes | Yes | Yes | Not Applicable | Not Applicable | Not Applicable |
| Markosyan 2014 [28] | Not Applicable | Not Applicable | Unclear | No | No | Unclear | Yes | Not Applicable | Not Applicable | Not Applicable |
| Muñoz 2013 [30] | Yes | Yes | Yes | Yes | Yes | Yes | Yes | Yes | Yes | No |
| Sardar 2015 [36] | Yes | Yes | Yes | No | No | Yes | Yes | Yes | Yes | Yes |
| Stalman 2015 [38] | Not Applicable | Not Applicable | Yes | Yes | Yes | Yes | Yes | Not Applicable | Not Applicable | Not Applicable |
| Stalman 2016 [39] | Not Applicable | Not Applicable | Yes | Yes | Yes | Yes | Yes | Not Applicable | Not Applicable | Not Applicable |
| White 2022 [42] | Not Applicable | Not Applicable | Yes | Yes | Yes | Yes | Yes | Yes | Yes | Yes |
| Yang 2010 [43] | Not Applicable | Not Applicable | Yes | Yes | Yes | No | Yes | Not Applicable | Not Applicable | Not Applicable |
| Yue 2019 [44] | Not Applicable | Not Applicable | Yes | Yes | Yes | Yes | Yes | Not Applicable | Not Applicable | Not Applicable |
| Items   1. Were the two groups similar and recruited from the same population? 2. Were the exposures measured similarly to assign people to both exposed and unexposed groups? 3. Was the exposure measured in a valid and reliable way? 4. Were confounding factors identified? 5. Were strategies to deal with confounding factors stated? 6. Were the groups/participants free of the outcome at the start of the study (or at the moment of exposure)? 7. Were the outcomes measured in a valid and reliable way? 8. Was the follow up time reported and sufficient to be long enough for outcomes to occur? 9. Was follow up complete, and if not, were the reasons to loss to follow up described and explored? 10. Were strategies to address incomplete follow up utilized? 11. Was appropriate statistical analysis used? | | | | | | | | | | |

| Table 7. Case series quality assessment (JBI checklist) – Clinical area | | | | | | | | | | |
| --- | --- | --- | --- | --- | --- | --- | --- | --- | --- | --- |
| Study | Item 1 | Item 2 | Item 3 | Item 4 | Item 5 | Item 6 | Item 7 | Item 8 | Item 9 | Item 10 |
| Machado 2012 [27] | Yes | Yes | Yes | Unclear | Unclear | Yes | Yes | Yes | Yes | Yes |
| Papadimitriou 2012 [31] | Yes | Yes | Yes | Yes | Yes | Yes | Yes | Not Applicable | Yes | Yes |
| Prasad 2012 [32] | Yes | Yes | Yes | Yes | Yes | Yes | Yes | Yes | Yes | Yes |
| Saengkaew 2017 [35] | Yes | Yes | Yes | Yes | Unclear | Yes | Yes | Not Applicable | Yes | Unclear |
| Items   1. Were there clear criteria for inclusion in the case series? 2. Was the condition measured in a standard, reliable way for all participants included in the case series? 3. Were valid methods used for identification of the condition for all participants included in the case series? 4. Did the case series have consecutive inclusion of participants? 5. Did the case series have complete inclusion of participants? 6. Was there clear reporting of the demographics of the participants in the study? 7. Was there clear reporting of clinical information of the participants? 8. Were the outcomes or follow up results of cases clearly reported? 9. Was there clear reporting of the presenting site(s)/clinic(s) demographic information? 10. Was statistical analysis appropriate? | | | | | | | | | | |

| Table 8. Diagnostic accuracy studies quality assessment (JBI checklist) – Clinical area | | | | | | | | | | |
| --- | --- | --- | --- | --- | --- | --- | --- | --- | --- | --- |
| Study | Item 1 | Item 2 | Item 3 | Item 4 | Item 5 | Item 6 | Item 7 | Item 8 | Item 9 | Item 10 |
| Lucio 2019 [25] | Unclear | Yes | Yes | Unclear | Unclear | No | Yes | Yes | Yes | Yes |
| Items   1. Was a consecutive or random sample of patients enrolled? 2. Was a case control design avoided? 3. Did the study avoid inappropriate exclusions? 4. Were the index test results interpreted without knowledge of the results of the reference standard? 5. If a threshold was used, was it pre-specified? 6. Is the reference standard likely to correctly classify the target condition? 7. Were the reference standard results interpreted without knowledge of the results of the index test? 8. Was there an appropriate interval between index test and reference standard? 9. Did all patients receive the same reference standard? 10. Were all patients included in the analysis? | | | | | | | | | | |

| Table 9. Cohort studies quality assessment (JBI checklist) – Biochemical area | | | | | | | | | | | |
| --- | --- | --- | --- | --- | --- | --- | --- | --- | --- | --- | --- |
| Study | Item 1 | Item 2 | Item 3 | Item 4 | Item 5 | Item 6 | Item 7 | Item 8 | Item 9 | Item 10 | Item 11 |
| Bhadada 2010 [46] | Yes | Yes | Yes | Yes | Yes | Yes | Yes | Not Applicable | Not Applicable | Not Applicable | Yes |
| Mali 2013 [50] | Yes | Yes | Yes | Yes | Yes | Yes | Yes | Not Applicable | Not Applicable | Not Applicable | Unclear |
| Sisley 2013 [51] | Not Applicable | Not Applicable | Yes | Yes | Yes | Yes | Yes | Not Applicable | Not Applicable | Not Applicable | Yes |
| Jawa 2016 [48] | Not Applicable | Not Applicable | Yes | Yes | Yes | Yes | Yes | Not Applicable | Not Applicable | Not Applicable | Yes |
| Essaddam 2019 [47] | Not Applicable | Not Applicable | Yes | Yes | Yes | Not Applicable | Yes | No | Yes | No | Yes |
| Items   1. Were the two groups similar and recruited from the same population? 2. Were the exposures measured similarly to assign people to both exposed and unexposed groups? 3. Was the exposure measured in a valid and reliable way? 4. Were confounding factors identified? 5. Were strategies to deal with confounding factors stated? 6. Were the groups/participants free of the outcome at the start of the study (or at the moment of exposure)? 7. Were the outcomes measured in a valid and reliable way? 8. Was the follow up time reported and sufficient to be long enough for outcomes to occur? 9. Was follow up complete, and if not, were the reasons to loss to follow up described and explored? 10. Were strategies to address incomplete follow up utilized? 11. Was appropriate statistical analysis used? | | | | | | | | | | | |

| Table 10. Cross-sectional studies quality assessment (JBI checklist) – Biochemical area | | | | | | | | |
| --- | --- | --- | --- | --- | --- | --- | --- | --- |
| Study | Item 1 | Item 2 | Item 3 | Item 4 | Item 5 | Item 6 | Item 7 | Item 8 |
| Lashari 2014 [49] | Yes | Yes | Yes | Yes | Yes | Yes | Yes | Yes |
| Xu 2019 [45] | Yes | Yes | Yes | Yes | Yes | Yes | Yes | Yes |
| Items   1. *Were the criteria for inclusion in the sample clearly defined?* 2. *Were the study subjects and the setting described in detail?* 3. *Was the exposure measured in a valid and reliable way?* 4. *Were objective, standard criteria used for measurement of the condition?* 5. *Were confounding factors identified?* 6. *Were strategies to deal with confounding factors stated?* 7. *Were the outcomes measured in a valid and reliable way?* 8. *Was appropriate statistical analysis used?* | | | | | | | | |

| Table 11. Excluded study – Clinical area | |
| --- | --- |
| Study | Exclusion criterion |
| Abawi, O., et al. (2021). "Impact of body mass index on growth hormone stimulation tests in children and adolescents: a systematic review and meta-analysis." Critical Reviews in Clinical Laboratory Sciences 58(8): 576-595. | Wrong study design |
| Abell, K., et al. (2016). "Fetal alcohol spectrum disorders and assessment of maxillary and mandibular arc measurements." Am J Med Genet A 170(7): 1763-1771. | Wrong outcome |
| Al Herbish, A. S., et al. (2016). "Diagnosis and management of growth disorders in Gulf Cooperation Council (GCC) countries: Current procedures and key recommendations for best practice." International Journal of Pediatrics and Adolescent Medicine 3(3): 91-102. | Wrong study design |
| Albareqy, A. H., et al. (2020). "AN OVERVIEW ON BARTTER SYNDROMES: LITERATURE REVIEW." Pharmacophore 11(5): 84-87. | Wrong outcome |
| Alharthi, A. A. (2016). "Idiopathic short stature in children: A hospital based study." Journal of Medical Sciences (Faisalabad) 16(3-4): 56-61. | Wrong population |
| Alyahyawi, N. Y. (2024). "Auxological, Clinical, and MRI Abnormalities in Pediatric Patients With Isolated Growth Hormone Deficiency." Cureus Journal of Medical Science 16(2). | Wrong population |
| Arnao, M. D. R., et al. (2014). "The DATAC study: A new growth database. Description of the epidemiology, diagnosis and therapeutic attitude in a group of Spanish children with short stature." Journal of Pediatric Endocrinology and Metabolism 27(11-12): 1201-1208. | Wrong population |
| Baig, U., et al. (2023). "Assessment of upper to lower body segment ratio and arm span to height difference in school children of Lahore." J Pak Med Assoc 73(5): 1043-1047. | Wrong population |
| Brusa, J., et al. (2020). "Comparison of Postural Features and Muscle Strength between Children with Idiopathic Short Stature and Healthy Peers in Relation to Physical Exercise." Sustainability 12(9). | Wrong population |
| Budzulak, J., et al. (2022). "Malnutrition as the cause of growth retardation among children in developed countries." Annals of Agricultural and Environmental Medicine 29(3): 336-341. | Wrong study design |
| Casaña-Granell, S., et al. (2021). "Adolescence and short stature: factors in adjustment to the diagnosis." Qual Life Res 30(8): 2275-2286. | Wrong population |
| Checkley, W., et al. (2024). "Effects of Cooking with Liquefied Petroleum Gas or Biomass on Stunting in Infants." New England Journal of Medicine 390(1): 44-54. | Wrong population |
| Christesen, H. T., et al. (2016). "Short Stature: Comparison of WHO and National Growth Standards/References for Height." PLoS One 11(6). | Wrong population |
| de Assis, P. P., et al. (2022). "Growth of infants with gastrointestinal manifestations of cow’s milk protein allergy." Revista de Nutricao 35. | Wrong outcome |
| De Cássia Carvalho Oliveira, F., et al. (2011). "Bolsa Família Program and child nutritional status: Strategic challenges." Ciencia e Saude Coletiva 16(7): 3307-3316. | Wrong outcome |
| del Pino, M., et al. (2018). "Leg length, sitting height, and body proportions references for achondroplasia: New tools for monitoring growth." American Journal of Medical Genetics Part A 176(4): 896-906. | Wrong population |
| Deng, S., et al. (2022). "Description of the molecular and phenotypic spectrum in Chinese patients with aggrecan deficiency: Novel ACAN heterozygous variants in eight Chinese children and a review of the literature." Front Endocrinol (Lausanne) 13: 1015954. | Wrong outcome |
| Elmighrabi, N. F., et al. (2023). "A systematic review and meta-analysis of the prevalence of childhood undernutrition in North Africa." PLoS One 18(4): e0283685. | Wrong outcome |
| Fink, G., et al. (2017). "Home- and community-based growth monitoring to reduce early life growth faltering: an open-label, cluster-randomized controlled trial." Am J Clin Nutr 106(4): 1070-1077. | Wrong outcome |
| Grimberg, A., et al. (2011). "Medically underserved girls receive less evaluation for short stature." Pediatrics 127(4): 696-702. | Wrong outcome |
| Harju, S., et al. (2022). "Epidemiology of Disorders Associated with Short Stature in Childhood: A 20-Year Birth Cohort Study in Finland." Clinical Epidemiology 14: 1205-1214. | Wrong population |
| Hawkes, C. P., et al. (2020). "Sitting Height to Standing Height Ratio Reference Charts for Children in the United States." J Pediatr 226: 221-227.e215. | Wrong outcome |
| Heemann, M., et al. (2021). "Assessment of Undernutrition Among Children in 55 Low- and Middle-Income Countries Using Dietary and Anthropometric Measures." JAMA Netw Open 4(8): e2120627. | Wrong outcome |
| Hoover-Fong, J. E., et al. (2021). "Growth in achondroplasia including stature, weight, weight-for-height and head circumference from CLARITY: achondroplasia natural history study-a multi-center retrospective cohort study of achondroplasia in the US." Orphanet J Rare Dis 16(1): 522. | Wrong population |
| Hussein, A., Farghaly, H., Askar, E., Metwalley, K., Saad, K., Zahran, A., & Othman, H. A. (2017). Etiological factors of short stature in children and adolescents: experience at a tertiary care hospital in Egypt. Therapeutic advances in endocrinology and metabolism, 8(5), 75-80. | Wrong population |
| Hussein, H. K., et al. (2023). "Association of baseline body mass index and other auxological data in short stature children with idiopathic growth hormone at Al-Rusafa district in Baghdad, Iraq." Rawal Medical Journal 48(3): 681-684. | Wrong outcome |
| Ji, Y. T., et al. (2022). "Body composition in preschool children with short stature: a case-control study." BMC Pediatr 22(1): 98. | Wrong population |
| Khadikar, V., et al. (2021). "Extended growth charts for Indian children." J Pediatr Endocrinol Metab 34(3): 357-362. | Wrong outcome |
| Kimani-Murage, E. W., et al. (2010). "The prevalence of stunting, overweight and obesity, and metabolic disease risk in rural South African children." BMC Public Health 10: 158. | Wrong population |
| Kondpalle, S., et al. (2019). "Upper and Lower Body Segment Ratios from Birth to 18 years in Children from Western Maharashtra." Indian J Pediatr 86(6): 503-507. | Wrong outcome |
| Maghnie, M., et al. (2018). "Short stature diagnosis and referral." Front Endocrinol (Lausanne) 8(JAN). | Wrong study design |
| Mali, L. P., et al. (2013). "Clinical and nutritional assessment of short statured Indian children with celiac disease." Journal of Nepal Paediatric Society 33(2): 91-94. | Wrong outcome |
| Markosyan, R. L., et al. (2012). "Children's normal and aberrant growth." New Armenian Medical Journal 6(2): 24-32. | Wrong study design |
| Marstrand-Joergensen, M. R., et al. (2017). "Prevalence of <i>SHOX</i> haploinsufficiency among short statured children." Pediatr Res 81(2): 335-341. | Wrong outcome |
| Martin, D. D., et al. (2011). "The use of bone age in clinical practice - part 1." Horm Res Paediatr 76(1): 1-9. | Wrong study design |
| Mehlman, C. T. and M. C. Ain (2015). "Evaluation of the Child with Short Stature." Orthop Clin North Am 46(4): 523-531. | Wrong study design |
| Nicholas, J. L., et al. (2020). "US Evaluation of Bone Age in Rural Ecuadorian Children: Association with Anthropometry and Nutrition." Radiology 296(1): 161-169. | Wrong outcome |
| Patel, R. and A. Bajpai (2021). "Evaluation of Short Stature in Children and Adolescents." Indian J Pediatr 88(12): 1196-1202. | Wrong study design |
| Patel, R., et al. (2021). "Predictive Value of IAP 2015, IAP 2007 and WHO Growth Charts in Identifying Pathological Short Stature." Indian Pediatr 58(2): 149-151. | Wrong study design |
| Rogol, A. D. and G. F. Hayden (2014). "Etiologies and early diagnosis of short stature and growth failure in children and adolescents." J Pediatr 164(5 Suppl): S1-14.e16. | Wrong study design |
| Sabinkar, G., et al. (2023). "Growth Velocity in South Indian Children Between Three and 18 Years of Age." Cureus Journal of Medical Science 15(12). | Wrong outcome |
| Sathyanarayanan, et al. (2022). "Different Cases of Short Stature." J Assoc Physicians India 70(4): 11-12. | Wrong study design |
| Savage, M. O., et al. (2016). "Early Detection, Referral, Investigation, and Diagnosis of Children with Growth Disorders." Horm Res Paediatr 85(5): 325-332. | Wrong study design |
| Savage, M. O. and H. L. Storr (2021). "Balanced assessment of growth disorders using clinical, endocrinological, and genetic approaches." Annals of Pediatric Endocrinology and Metabolism 26(4): 218-226. | Wrong study design |
| Scherdel, P., et al. (2016). "Growth monitoring as an early detection tool: a systematic review." Lancet Diabetes & Endocrinology 4(5): 447-456. | Wrong population |
| Scherdel, P., et al. (2013). "Growth Monitoring: A Survey of Current Practices of Primary Care Paediatricians in Europe." PLoS One 8(8). | Wrong study design |
| Sheikhi, V., Bonyadi, S., & Heidari, Z. (2022). Causes of short stature in children referred to a tertiary care center in Southeast of Iran: 2018-2020. Journal of Pediatrics Review, 10(1), 73-82. | Wrong population |
| Surana, V., et al. (2018). "Short stature - Clinical approach to diagnosis: A 2018 perspective." Journal of the Indian Medical Association 116(10): 56-62. | Wrong study design |
| Taylor-Miller, T. and P. J. Simm (2017). "Growth disorders in adolescents." Aust Fam Physician 46(12): 913-917. | Wrong study design |
| Valdes, A., et al. (2018). "The Short Child." Pediatr Ann 47(1): e29-e35. | Wrong study design |
| van Dommelen, P. and S. van Buuren (2014). "Methods to obtain referral criteria in growth monitoring." Statistical Methods in Medical Research 23(4): 369-389. | Wrong study design |
| van Dommelen, P., et al. (2021). "Guideline for referring short or tall children in preventive child health care." Acta Paediatr 110(4): 1231-1238. | Wrong study design |
| Vlaardingerbroek, H., et al. (2024). "Assessment of Nutritional Status in the Diagnostic Evaluation of the Child with Growth Failure." Horm Res Paediatr 97(1): 11-21. | Wrong study design |
| Wahyuningsih, H. P., et al. (2020). "Scoring model using stunting cards for toddlers." Pakistan Journal of Medical and Health Sciences 14(2): 1419-1424. | Wrong outcome |
| Wit, J. M., et al. (2019). "Towards a Rational and Efficient Diagnostic Approach in Children Referred for Growth Failure to the General Paediatrician." Horm Res Paediatr 91(4): 223-240. | Wrong study design |
| Xiong, F., et al. (2014). "Epidemiological investigation of physique situation for birth high-risk children aged 9-15 years in Chengdu, Southwest China." Clin Exp Obstet Gynecol 41(1): 52-57. | Wrong study design |
| Yadav, S. and A. Dabas (2015). "Approach to short stature." Indian J Pediatr 82(5): 462-470. | Wrong study design |
| Yadav, A. K., et al. (2020). "A Hospital Based Study of Short Stature among children in India from the Eastern UP Population." European Journal of Molecular and Clinical Medicine 7(10): 2607-2619. | Wrong study design |

| Table 12. Excluded study – Biochemical area | |
| --- | --- |
| Study | Exclusion criterion |
| Assiri, A. M. A. (2010). "Isolated short stature as a presentation of celiac disease in Saudi children." Pediatric Reports 2(1): 15-17. | Focus no short stature |
| Bibi, A., Aamir, M., Haroon, Z. H., Maqsood, U., & Qamar, U. (2023). Etiological study of short stature in children and role of insulin like growth factor-1 and insulin like growth factor binding protein-3 as screening markers for growth hormone deficiency. JPMA. The Journal of the Pakistan Medical Association, 73(2), 323-327. | Wrong Biochemical Roles |
| Blum, W. F., et al. (2018). "The growth hormone-insulin-like growth factor-I axis in the diagnosis and treatment of growth disorders." Endocrine Connections 7(6): R212-R222. | Wrong study design |
| Bueno, A. L., et al. (2010). "Calcium and vitamin D intake and biochemical tests in short-stature children and adolescents." Eur J Clin Nutr 64(11): 1296-1301. | Wrong Biochemical Test |
| Cavarzere, P., et al. (2024). "Role of genetic investigation in the diagnosis of short stature in a cohort of Italian children." J Endocrinol Invest 47(5): 1237-1250. | Wrong Biochemical Roles |
| Collett-Solberg, P. F., et al. (2019). "Diagnosis, Genetics, and Therapy of Short Stature in Children: A Growth Hormone Research Society International Perspective." Horm Res Paediatr 92(1): 1-14. | Wrong study design |
| Corripio-Collado, R., et al. (2022). "Delphi consensus on the diagnosis and treatment of patients with short stature in Spain: GROW-SENS study." J Endocrinol Invest 45(4): 887-897. | Wrong study design |
| Dori, E. B., et al. (2020). "The inter - Test variability of growth hormone stimulation tests and factors affecting this variability." Growth Horm IGF Res 55: 101361. | Focus no short stature |
| Hussein, A., Farghaly, H., Askar, E., Metwalley, K., Saad, K., Zahran, A., & Othman, H. A. (2017). Etiological factors of short stature in children and adolescents: experience at a tertiary care hospital in Egypt. Therapeutic advances in endocrinology and metabolism, 8(5), 75-80. | Wrong Biochemical Roles |
| Laczmanska, I., et al. (2018). "Short stature in genetic syndromes: Selected issues." Advances in Clinical and Experimental Medicine 27(3): 409-414. | Wrong Biochemical Test |
| Léger, J. (2017). "How should we investigate children with growth failure?" Annales D Endocrinologie 78(2): 106-107. | Wrong study design |
| Leung, A. K. C. and A. A. C. Leung (2018). "Evaluation and management of short stature in children." Consultant 58(8): 195-208. | Focus no short stature |
| Maliachova, O., et al. (2019). "Cortisol Levels in Glucagon Stimulation Test in Children Assessed for Short Stature: Clinical and Laboratorial Correlations." Horm Metab Res 51(12): 798-804. | Wrong Biochemical Test |
| Mehlman, C. T. and M. C. Ain (2015). "Evaluation of the Child with Short Stature." Orthop Clin North Am 46(4): 523-531. | Wrong Biochemical Roles |
| Polidori, N., et al. (2020). "Deciphering short stature in children." Annals of Pediatric Endocrinology & Metabolism 25(2): 69-79. | Wrong Biochemical Roles |
| Rashid, A., et al. (2022). "Assessment of Vitamin D Status and Growth Parameters in Thalassemia Major Children." Pakistan Paediatric Journal 46(4): 429-433. | Focus no short stature |
| Sheikhi, V., Bonyadi, S., & Heidari, Z. (2022). Causes of short stature in children referred to a tertiary care center in Southeast of Iran: 2018-2020. Journal of Pediatrics Review, 10(1), 73-82. | Wrong Biochemical Roles |
| Singh, H., et al. (2018). "Application of chromosomal microarray for evaluation of idiopathic short stature in Asian Indian children: A pilot study." Indian Journal of Endocrinology and Metabolism 22(1): 100-106. | Wrong Biochemical Roles |
| Sodero, G., et al. (2023). "Growth hormone responses during arginine and clonidine stimulation test: Correlations with patients' auxological and metabolic parameters in a single centre study." Growth Horm IGF Res 68: 101522. | Wrong Biochemical Test |
| Sun, M., et al. (2020). "Association between serum calcium and phosphorus levels and insulin-like growth factor-1 in chinese children and adolescents with short stature." International Journal of General Medicine 13: 1167-1173. | Wrong Biochemical Roles |
| Tenenbaum, A., et al. (2014). "The intramuscular glucagon stimulation test does not provide good discrimination between normal and inadequate ACTH reserve when used in the investigation of short healthy children." Horm Res Paediatr 82(3): 194-200. | Wrong Biochemical Roles |
| Thakur, D. S., et al. (2018). "Clonidine stimulation test: Is single best time point, convenient yet efficacious?" Indian Journal of Endocrinology and Metabolism 22(4): 511-514. | Wrong Biochemical Roles |
| Thieme, F., et al. (2022). "The influence of body mass index on the growth hormone peak response regarding growth hormone stimulation tests in children." Horm Res Paediatr 95(5): 452-460. | Wrong Biochemical Test |
| Weintrob, N., et al. (2018). "SERUM FREE CORTISOL DURING GLUCAGON STIMULATION TEST IN HEALTHY SHORT-STATURED CHILDREN AND ADOLESCENTS." Endocr Pract 24(3): 288-293. | Wrong Biochemical Roles |
| Yau, M. and R. Rapaport (2022). "Growth Hormone Stimulation Testing: To Test or Not to Test? That Is One of the Questions." Front Endocrinol (Lausanne) 13: 902364. | Wrong Biochemical Roles |
